# Veterinary professional identity: Conceptual analysis and location in a practice theory framework

**DOI:** 10.3389/fvets.2023.1041475

**Published:** 2023-02-09

**Authors:** Emma Scholz, Franziska Trede

**Affiliations:** ^1^School of Animal, Environmental and Veterinary Sciences, Charles Sturt University, Wagga Wagga, NSW, Australia; ^2^Institute for Interactive Media and Learning, University of Technology Sydney, Sydney, NSW, Australia

**Keywords:** professional identity, veterinary practice, practice theory, sociocultural theory, dialogue

## Abstract

Professional, social, and cultural issues and phenomena of veterinary practice are now established areas of commentary and interest in research, education, professional publications and even in the mainstream media. Despite the availability of theoretically informed literature in diverse relevant domains and disciplines including professional practice, workplace learning, and medical sociology and anthropology, commentary and research on veterinary practice issues and phenomena remains dominated by clinician-educators and clinician-policymakers. Reflecting the clinical disciplinary traditions, there is a resulting over-representation of individualistic, positivist perspectives and under-theorized research studies. In this paper we provide an interdisciplinary theoretical framework for veterinary practice and veterinary professional identity grounded in a practice theory perspective. We begin by arguing for the need for such a framework by scoping veterinary practice in its contemporary social context. We go on to provide a sociocultural framing of veterinary practice, underlining the mutual constitution of individuals and the social world through participation in practices and taking into consideration important concepts including knowledge, institutions, ethics, and embodiment. We assert the importance of professional identity as a core phenomenon of veterinary practice, constituted by making meaning of professional practice experiences, especially through narrative and dialogue. This practice theory framework for veterinary practice and veterinary professional identity development provides rich opportunities for understanding, researching, and enacting diverse activities and phenomena, especially learning, development and change within and beyond formal educational settings.

## 1. Introduction

In this paper, we offer a narrative conceptual framework grounded in sociocultural and practice theories for understanding and exploring veterinary professional identity as a core dimension of veterinary practice. The analysis is contextualized at different levels, beginning by characterizing contemporary societal conditions and then focusing on veterinary practice specifically, exploring issues that impact on veterinarians.

There are multiple reasons to value a detailed articulation and a rich conceptualization of veterinary professional identity, and here we mention three. Firstly, when a term like identity is used casually and repeatedly in everyday life, it can take on an all-encompassing character that paradoxically strips it of meaning. Providing a conceptually rigorous and coherent account of identity as a phenomenon makes a strong case for placing professional identity at the heart of veterinary practice, restoring its importance and worthiness of resourcing to explore and develop. Secondly, this framework points to novel research questions to pose, and innovative research approaches that can fruitfully explore veterinary practice and its associated dimensions as human activities grounded in specific times and places. Others may be emboldened to explore diverse disciplines for other conceptual frameworks that can provide useful insights. Finally, a careful theoretical conception provides tools with which to rigorously and critically evaluate and explore claims or interventions that purport to impact on professional identity and its development within and beyond formal educational settings.

We begin the paper by contextualizing our analysis with an outline of contemporary social conditions and how they impact on veterinarians. We then provide a sociocultural practice theory perspective of veterinary practice, drawing on the theory of practice architectures to underline the mutual constitution of individuals and the social world through participation in practices and taking into consideration important concepts including knowledge, institutions, ethics, and embodiment. Making meaning of professional practice experiences is highlighted as a crucial dimension of being a veterinarian, and the way in which professional identity is made visible and developed. We highlight narrative and dialogue as key cultural tools through which veterinarians make meaning of their experiences, enable agency, and author themselves.

## 2. Veterinary practice in a changing world: Being veterinarians in conditions of supercomplexity

Veterinary practice is associated with health, welfare, production, and performance in a diverse range of nonhuman animal species across the globe. It occurs in the ubiquitous interface between humans and animals, being linked with domestication in a wide variety of species for different purposes such as food, fiber, labor, economic security, entertainment and companionship. Contemporary global conditions have been characterized as comprising *supercomplexity*, reflecting unprecedented change and uncertainty (1, 2). Supercomplexity constitutes a qualitative distinction from conditions of complexity. Under complexity, sufficient resources can solve problems that arise, whereas supercomplexity is characterized by competing and incommensurable frameworks for understanding (1). That means that even if infinite resources were available, problems associated with supercomplexity would not be amenable to resolution. The defining conditions for supercomplexity are described as contestability, challengeability, unpredictability, and uncertainty. They signpost that we have given up on control and predictability. Professions, including medicine, have suggested they function in an age of complexity, uncertainty, and reflection (3). Under those conditions, professionalism is challenged by competing discourses of managerialism, entrepreneurialism, and consumerism and there are calls for criticality and creativity in order to retain independence, standards, and an ethical grounding (2). Veterinarians, collectively and individually, perceive the impacts of supercomplexity through their experience of changes that include advances in technologies and knowledge, demographic and political shifts, globalization of agricultural markets, and environmental and climatic changes.

In the following section, we draw attention to some of the contemporary issues with which the veterinary profession is concerned, based on scholarly literature and commentary within the profession. There is a predominance of voices from developed nations, particularly North America, Europe, the United Kingdom and Australia. Global influences impact veterinarians in all parts of the world, but we acknowledge that there is much yet to be understood and heard from under-researched colleagues in developing nations.

### 2.1. Professionalization: An ongoing struggle for position and reward

Professionalization refers to ways in which an occupational group, in this case veterinarians, comes to see themselves and to be accepted by the community as a profession. The emergence of professions in general, and the veterinary profession in particular, can be linked to wider global trends associated with the development of the institutions and structures of modernity, including the development of nation-states, the rise of the rule of law, and the evolution of financial instruments (4). For those involved with animal health, specific processes of agricultural and economic development, property rights, concerns with public health, and changing legal and cultural status of animals also played a major role (5, 6). Professionalization represents the process by which a group comes to recognize itself and be recognized by its community as having some coherence, by means of shared ways of understanding, standards, and responsibilities (7). Processes of professionalization trace diverse trajectories across different geographical, political and cultural settings. Being accepted as a profession carries significant benefits such as public status, economic reward, claims to self-determination and trustworthiness, which appears to be a designation to be pursued and guarded assiduously (4). Veterinary history, as told from within, can be cast as a steady advance, with inevitable and smooth progress through stages marked by the work of founding fathers—who were, until very recent years, mostly male with a few notable exceptions—in creating structures and institutions, and in advancing knowledge (8, 9). Alternative accounts present a struggle for recognition, status, and reward in which powerful countervailing voices, chance events, and human frailties all played their roles (5, 10).

In developed economies, professions and economics make somewhat uncomfortable bedfellows, with one of the features of achieving the status of a profession in the eyes of the community being the expectation that professionals will place their duty to their clients or patients above their own financial interests. On the other hand, there is a reciprocal expectation that professionals will be rewarded for that trust, both financially and in terms of prestige and autonomy. Veterinarians perceive a significant mismatch between their clients' perceptions of high veterinary costs and their own experience of modest financial reward for long hours of often arduous work, expectations of competency across multiple species, frequently high levels of study debt, and responsibility to provide 24-h emergency care (11–13). The economic tensions alive within the profession are linked in complex ways to ideas about professionalism but also to community understandings of responsibility for animals' welfare and health in circumstances of changing human–animal relations. There is a tension between community desires for animal welfare to be prioritized and the lack of a social safety net when the user-pays system fails.

### 2.2. Changing human-animal relations

Development of practices associated with animal survival, health, production, and performance follow changes in the conduct of human social life. Utilization by humans confers a value on animals, whether material, economic, cultural, or emotional, and notions of ownership or stewardship locate responsibility for husbandry tasks (14, 15). The human- animal bond is an ancient and established feature of human animal relations, but its characteristics change as societies change. In the contemporary developed world, veterinarians' responsibilities toward animals are located at the interface of animal welfare science, codified regulatory frameworks, and ethical considerations about responsibilities to other people and the moral status of animals. As a result, veterinarians go about their daily work in a complex and contested philosophical landscape with concrete implications for their practice (16–18).

Mounting empirical evidence of animal sentience and intelligence supports a philosophical view of veterinary ethics as a focus of conflicting interests (18), with animals and humans each having legitimate interests and moral claims that are not easily balanced. In developed countries, an increasingly urbanized population with unprecedentedly high standards of living but experiencing disturbing levels of loneliness (19) has fuelled an increase in companion animal ownership. At the same time, urban populations are almost entirely unconnected with the livestock industries that produce their food, a phenomenon that has been described as “post-domestication” (20), meaning that the majority of the general population have little to no direct experience with, or understanding of, farming practices. The closeness and love that people feel toward their pets supports a discourse that portrays companion animals as part of the family (21, 22). There are economic opportunities for veterinarians in harnessing the willingness of companion animal owners to invest in health care for pets, an investment that may extend to costly interventions, preventative health care, and extensive end-of-life care. At times of pressure, however, there remain significant inconsistencies in people's behavior and attitudes toward animals, both individually and at a community level (23). Regulations governing veterinary practice and animal management, as well as broader community norms and expectations, create conditions in which veterinarians are required to balance human and animal interests in specific situations and advise and enact appropriate courses of action.

### 2.3. Wellness challenges for veterinarians and veterinary students

A plethora of evidence in recent decades points to serious problems of wellness within the veterinary profession (24–28). Rates of mental distress in practicing veterinarians have been reported as being significantly higher than in the general population. Mental distress includes measures of anxiety, depression, burnout, suicidal thoughts, and substance abuse (29–31). At the extreme end, suicide rates for veterinarians are globally higher than those in the general population, and also higher than in other high-risk professions (26, 32, 33). Professional institutions and the community are paying attention, with high profile campaigns from professional associations to address profession-wide issues of poor mental health (34, 35). Complex relationships between individual characteristics, environmental conditions, stressors, and protective factors mediate wellbeing for veterinarians and veterinary students (25, 29, 36–38). Commonly cited reasons for distress in veterinary students and veterinarians include interrelated issues such as high workload, fear of failure or error, long and arduous hours of work and the emotional cost of routinely performing euthanasia (32, 39, 40). Perceived stigma is reported to be a barrier to effective help-seeking for mental health challenges in veterinarians and veterinary students (24, 32), a finding also noted in the medical profession (41). The perception of stigma is not necessarily irrational in an environment where the concept of “fitness to practice” may be interpreted or operationalised in overly rigid terms as complete freedom from impairment (42, 43). Although limited outcomes-based evidence is available when it comes to strategies for supporting wellbeing in veterinarians and veterinary students (44), there is a place for rich and informative research to explore the intertwinements of people with their practice and educational contexts. Researchers are beginning to uncover deeply held beliefs and expectations among veterinarians about belonging, perfectionism, uncertainty, and error (36, 45–49).

### 2.4. Gender in a “feminizing” veterinary profession

The veterinary profession remained numerically male dominated until well into the second half of the twentieth century, but demographic change occurred rapidly and has been a global phenomenon. By the late 1980s, 50% of undergraduate veterinary students were women in Australia and the USA and they now comprise 80% of veterinary graduates, a phenomenon that appears to be similar across Europe, North America and Australia (50–52). This change in the gender balance has been the subject of considerable debate and commentary within the profession. Overt expressions of sexism are no longer acceptable, but a continued undertone of ambivalence can be detected. Commentators and authors of opinion pieces have speculated about whether increasing numbers of women veterinary graduates represent challenges for the profession, linked with difficulties in recruitment outside metropolitan areas, with the relatively low level of remuneration for veterinarians, or with the economic impact of mental health problems (53–57). The ongoing position of women in the veterinary profession has recently been characterized as a post-feminist paradox, an experience of career limitation at odds with a rhetoric of unconstrained opportunity (58).

In discussing the increased numbers of women in the veterinary profession in Australia and elsewhere, the term “feminisation” has been used (59, 60), but it has been suggested that feminisation in the veterinary profession can actually serve to reinforce and perpetuate gendered assumptions and traditional gender roles and position both women and men accordingly (53, 61–64). As an example, the commonly espoused strengths of female veterinarians in relational and caring qualities can be positioned as being inimical to qualities required for economic success and career enhancement (65). In spite of the fact that women have contributed at least equally to graduate numbers for around three decades, they continue to be significantly underrepresented in senior and leadership roles (66–69). The presence and extent of a gender pay gap in the veterinary profession has been repeatedly demonstrated, although its existence remains contested (51, 56, 70–73). Some deny the existence of a gender-based “gap”, instead framing differences in terms of individual choices over time about working hours, career breaks, and practice ownership (70).

Current debate and discourse about the challenges and opportunities for veterinarians, and projecting to the future can be framed in various ways by different stakeholders. We have highlighted four socio-cultural elements that shape veterinary practice: professionalization, changing human-animal relations, wellness, and gender issues. Institutions such as professional associations, registering bodies, and veterinary schools have their own interests to promote and defend. As veterinarians make their way in conditions of supercomplexity, research is needed that brings individual practitioners and contextual factors into one carefully theorized frame.

## 3. Locating veterinary professional identity in a sociocultural practice theory frame

### 3.1. Social practice theory and professional practices

In this paper, we outline one conceptual framework for exploring veterinary professional practice and identity, based on concepts from the broad and diverse orientations to human social activities that come under the umbrella of “practice theory.” Practice theory may be best considered as a family of theoretical orientations encompassed by a disparate group of scholars (74–79), all of whom engage with the notion of how social practices organize and shape human life and activity. One thread that runs through the work of practice theorists is the effort to reach a “holistic way of thinking that integrates what people do, where they do it, with whom and for what purpose” (80). To engage with social practice theory, we need to draw on concepts that go beyond the dominant focus that has been described as being on “what is in the heads of individual practitioners” (77).

The theory of practice architectures is one conceptual account of professional practice; Kemmis and Grootenboer (81) draw on the work of philosophers of practice including Schatzki (79, 82) and Macintyre (78). In this account, the activities of practices comprise:

- *Sayings*, or activities in the cultural-discursive domain: overt or unspoken understandings about knowledge, about how things are, or should be done, and how to understand and be understood.
- *Doings*, or activities in the material-economic domain: actions and relations involving human or non-human bodies, objects, physical artifacts like computers, surgical instruments or buildings, and abstract concepts like money.
- *Relatings*, or activities in the social-political domain: actions involving relationships with other people including clients, other individual veterinarians, communities of practice such as the profession collectively. Relationships are always mediated by power relations.

Each of these domains is comprised of mutually constituted individual and extra-individual dimensions. Individual dimensions represent opportunities for agency, but agency that is always bounded by the mediating preconditions that the practice provides. The extra-individual dimensions are the practice architectures, from which the theory is entitled. The notion of practice architectures suggests vivid images of walls and doorways, good and poor design, and unexpected outcomes that may not have appeared in the original plan. Particular practice architectures may be very stable and long-lasting, or they may be ephemeral and dependent on fast-changing contextual conditions. Either way, they represent the mediating preconditions in which practice is enacted; The actions of any individual practitioner are guided and prefigured, although not wholly determined, by the practice architectures they encounter (81). If that were the case, there would be no human agency, and choice and responsibility would be terms shorn of meaning. Nonetheless, the modesty of possibilities for agency reminds us that changing professional practice requires changes “outside the heads” (83) of individual practitioners. Those changes include changes to the discourse in and of the practice, changes to the relationships enacted and sustained within the practice, and changes to the material and economic arrangements under which the practice is carried on. Such change is scarcely possible when professional practice is framed and researched within a purely individual frame.

In Table 1 we provide a list of some key features of professional practice viewed through the lens of practice architectures. These features can also be used as conceptual tools.

**Table 1:** Key features of professional practice.

**Feature**	**Description**
Professional practice is always relational	Relationality is more than the importance of interpersonal relations and communication and interactions and reflects something more profound about the intentionality of all sayings, doings, and relatings. When practitioners speak, they speak to, about, in response to, and in anticipation of, something and someone. Intentionality can occur across time and space, and even within the self.
Professional practice has meaning and purpose	Meaning and purpose are attached to all the sayings, doings and relatings of veterinary practice; actively making meaning is an important professional activity.
Professional knowledge and ethics are intertwined	Notions of what is good are at the heart of making decisions about what is right (Macintyre). Rich accounts of knowing and practice include practical reasoning which is “pragmatic, variable, context-dependent, and oriented toward action” [(84), p. 2].
Professional practice is situated, temporal and embodied	Without abandoning abstract notions such as competence, evidence, or professionalism, a rich understanding of veterinary practice demands deliberate exploration of how actual people do tasks involving other people and animals in specific settings, with the time they have available, and using the tools they can muster.

Using the theory of practice architectures, the deep connection between professional practice and professional identity can be made explicit. When veterinarians make decisions, act, and account for their actions to themselves and others in the course of their daily practice, they are engaging in veterinary practice. Sometimes with purpose and deliberation, but at other times unreflectively or through habit, they are also engaging with what it means to be themselves and also to be veterinarians and their engagement is therefore a matter of professional identity.

### 3.2. Understanding identity

Identity is a concept that draws on diverse disciplinary traditions such as philosophy, psychology, anthropology, sociology, and cultural studies. These disciplines overlap and share concepts, but they have distinct concerns, norms, and ways of coming to understand the world (85–88). Furthermore, identity is also an everyday word that does significant, and usually unnoticed, cultural work. We are named, grouped, described, and evaluated by ourselves and by others as a matter of course. The unity understood to comprise people and their identity or identities can sit uncomfortably with the diverse, even conflicting, roles and contexts in which human beings live. People navigate their way through settings in which their identity is framed very differently in terms of profession, gender, ethnicity, role, interests, political commitments, and geography. In this paper, we draw to a significant extent on a sociocultural theoretical framing of identity as a dialogical and developmental phenomenon (89), a framing that is conceptually aligned with the professional practice perspective above.

*People tell others who they are, but even more important, they tell themselves and then try to act as though they are who they say they are. These self- understandings, especially those with strong emotional resonance for the teller, are what we refer to as identities* (89).

Holland et al. (89) provided concepts that inform important dimensions of identity: the complexity of a self that is inward-facing but at the same time only has meaning in relation to others, the possibilities and limitations of human agency, the need to organize and confer meaning on experience using language, and the critical significance for people of the processes and discourses of identity. Holland et al.'s framing of identity blends power relations and discourses with creating a space for agency in developing their self-owned identity.

#### 3.2.1. The relational nature of self

Identity reflects the ways in which people name, characterize, and understand themselves and one another within, and in relation to, the social world. It is always entwined with selfhood and what it means to be a self, but “one is a self only among other selves. A self can never be described without reference to those who surround it” (90). People talk, they sing, they read, they play games, and fight and do business, as well as think. All those activities are identity-shaping and at their core social and relational. Those activities shape and develop people's identities, mediated through ideas, language, and power (87, 91). The self develops by means of drawing on socially derived discourses and is embedded in diverse social practices, with a relational self “developing at an interface, within the interplay between the social and embodied sources of the self, in what might be called the self-in-practice” (89).

#### 3.2.2. Mediated agency

From a sociocultural, practice-based perspective, the mutual constitution of the individual and the social means that possibilities for agency are mediated by means of sociomaterial setups, through cultural norms, through individual dispositions, and through complex interrelationships of some or all of those elements (89). The individualistic rhetoric of professional practice places emphasis on individual practitioner agency through use of terms such as autonomy and competence and does not align well with the notion of mediated agency. The modesty of the possibilities for individual agency in specific practice situations is not apparent and can be experienced as a surprise to those who subscribe to a sense of agency that is individualistic and heroic.

Professional practitioners bring their own unique combination of experiences, dispositions, and capabilities, and they encounter practice situations that are novel and specific. There is much that they are unable to influence but understanding agency as mediated or constrained constitutes a reminder to be aware, curious, and alert to opportunities for agency, or “pools of autonomy” (2) as they present themselves.

#### 3.2.3. Identity as making meaning

The activity of making meaning is held to be fundamental to what it is to be human (78, 92, 93) as people arrange and reconstruct experiences, objects, actions, and relations using the understandings and resources they can draw on to make sense of themselves and their world. Through linking narrative with self-understanding, scholars have made the case that making meaning through narrative is a way of developing and sustaining identity (88, 94, 95). Narrative makes stories of experience(s); whether shared with others processed inwardly, experience is organized and recast. Discourse and shared narrative resources play an important role in narrative. A dialogic perspective on identity provides a space for people to make meaning about themselves as well as the world through finding ways to use available resources and strategies. They can therefore author themselves as they author the world, making identity development simultaneously inward- and outward-facing (89). Self-authoring is dialogic, embodied, and embedded in social practices, as the individual develops an internalized sense of the responses and social judgements of others. The space for agency in enabling critique and improvisation in practice is a critical identity development process.

#### 3.2.4. Moral dimensions of identity

Framing identity in explicitly moral terms carries implications; Taylor asserts that knowing oneself is a question of *knowing where I stand. My identity is defined by the commitments and identifications which provide the frame or horizon within which I can try to determine from case to case what is good, or valuable, or what ought to be done* (90).

Identity is presented here as a matter of great consequence for people, with the potential to be a frame that supports important judgements about what is good and right, and what constitutes the good life. The evaluative dimension of the development of identity means that people make choices about what they value and seek to pursue. In doing so, and in making meaning of those choices, they spin their life story into a continuous thread. That thread was expressed by MacIntyre when he stated that “generally to adopt a stance on the virtues will be to adopt a stance on the narrative character of human life” (78) as a central idea in his moral theory.

#### 3.2.5. Understanding identity in dialogical terms

Although we remain the same person, identity develops and changes through life experiences, creating a tension between continuity and change. Human life is lived, commitments are made, and responsibilities are assigned based on the understanding that “we have to be able to respond to the imputation of strict identity. I am forever what I have been at any time for others” (78). Nonetheless, the possibility of transformation, of becoming something or someone new through desire or necessity, entails emergent notions of identity. Abandonment of either the essentialist perspective or the possibility of transformation seems to entail an impoverishment of the human experience.

*Humans are both blessed and cursed by their dialogic nature—their tendency to encompass a number of views in virtual simultaneity and tension, regardless of their logical incompatibility* (89).

A sociocultural perspective allows the location of identity in more than one space and asserts that it is necessary to reject neither traditional “essentialist” views of identity nor constructivist perspectives that locate an ever-changing identity in discourse communities. A dialogical and developmental practice theory of self and identity has identity emerging in the individual through interaction with others in the cultural and material world. Dialogue is also to be understood as a means for understanding the relational self so that identity allows for multiple sites of the self, a self that is grounded in social practices. Framing identity in dialogical terms allows for incoherence and instability to sit alongside unity and continuity (89, 96). Conceiving identity as being at once collective and individual is consistent with our framing of veterinary practice.

## 4. Professional identity in the veterinary and medical professions

In this section, we explore what is known about professional identity development within the veterinary profession. The veterinary scholarly professional identity literature is limited and relatively under-theorized; we also draw on the larger and longer-standing body of literature in the medical profession. Veterinarians share common ground with medical doctors in ways that have resonance for professional identity. The underpinning scientific knowledge of the structure and function of bodies; the techniques, medicines, equipment, and terminology of the clinic; and the discourse of health and illness present clear biomedical parallels between the veterinary and medical settings. The privilege and responsibility borne by the individual practitioner for patients is analogous between human and veterinary medical practitioners. The medical practice literature has matured in the last four decades to include socio-cultural elements, paying attention to the specific and unique dimensions that impact and influence veterinarians. A strong theme in professional identity research reflects a sense of fast-changing societal and political conditions that provide challenges to professions and professionals, consistent with previous descriptions of contemporary social conditions as constituting supercomplexity (2). Such influences have impact on government policy and societal norms, but their effects are also felt down to the level of workplace relationships and personal professional choices. Professional identity research that focuses on conditions of social change may reflect a sense of vulnerability and threat, or alternatively a challenge to entrenched positions of privilege and power. Integrating the social, environmental and cultural into professional identity research advances professions into the future because the future is inter-, multi- and transdisciplinary (97).

### 4.1. Professional identity in veterinarians

In the veterinary profession, professional identity is beginning to emerge as a topic of research interest (98), with the most sustained scholarly engagement being that of Armitage-Chan and her colleagues. Armitage-Chan focuses on professional identity with a goal of informing undergraduate veterinary curriculum development, espousing the position that a curriculum shaped around professional identity development promises to support increased career satisfaction and better mental health in the veterinary profession (95–100). Her research involved new graduate veterinarian participants and adopted a narrative inquiry methodology, framing professional identity in psychological terms, representing how a combination of individual veterinarian's moral views, professional priorities, and values were mobilized in making decisions and acting in veterinary practice. Armitage-Chan and May (99, 100) distinguished two modes of professional identity in which their participants recounted practice experiences. The first, and one that dominated during their very early practice experiences, was a diagnosis-focused identity in which technical features of patients and their illnesses dominated the accounts. The second mode was framed as a challenge-focused professional identity, in which technical competence remained salient, but contextual dimensions of practice were specifically included in the narrative as part of the experience and a potential source of satisfaction. Interestingly, Armitage-Chan and May proposed that the diagnosis-focused identity was modeled on an academic clinician role model previously identified within the medical profession (101) and that it was problematic to align with the circumstances of the general practice work setting for most graduates. Armitage Chan and May went on to develop a model and resources for curriculum development based around development of what they presented as a preferred identity, the challenge-focused identity (102, 103).

It is promising to see advocacy for explicit inclusion of professional identity development within the veterinary university curriculum. The notoriously crowded curriculum can be overly dominated by clinical knowledge and technical skills, and outcomes-based curricula are awkward spaces for the inclusion of inherently developmental phenomena. The psychological framing of professional identity as described by Armitage-Chan is one valid way of framing this notoriously slippery concept. However, the limitations of individualistic perspectives on phenomena like professional identity are that extra-individual dimensions of the professional world–economic structures, power relationships, traditions–are decentred and appear as the backdrop against which individuals act. The responsibility on the individual veterinarian to manage, understand, manage, and even overcome their context seems heavy indeed. We argue strongly for the inclusion of perspectives that balance individual agency with sociocultural structures and norms.

Perrin (104) explored professional identity development in veterinary students and new graduates in the United Kingdom. She posited associations between matters of professional identity and mental health and wellness challenges for veterinarians to argue for the existence of mismatches and dissonance between institutional rhetoric and lived experience of and about veterinary graduates and the nature of veterinary practice. We offer two observations of Perrin's work: First, although the institutions consistently portrayed the veterinary profession in terms of scientific rigor and clinical skill, the novices expressed their practice model in terms of care and vocation. Second, this research identified contradictions apparent in the institutional literature about the capabilities of new graduate veterinarians as representing a “dichotomy between viewing newly-qualified vets as being omnicompetent on graduation, set against viewing them as almost dangerously inept and in need of serious supervision” (104). Both the tradition of the hard-working veterinarian and the dissonances between rhetoric and reality were suggested as possibly being involved in the well-documented issues around wellness and mental health in the profession. Perrin was critical of institutional rhetoric that promotes a view of veterinary practice that is not well aligned with the goals and values of the profession's junior members, although her acceptance of official publicly-facing documents as a mirror of institutional and professional culture fails to account for the increasingly well-recognized influence of the hidden curriculum (105).

Illustrating the power of language, one study focused on the capacity for identity as a notion to be adapted to different interests and purposes. Framing the topic as exploring career identity in the veterinary profession, the author began, “The veterinary industry is transforming” (106). Use of the terms *career* and *industry* in place of *professional* and *profession* signaled that the authors located themselves in the discipline of organizational studies, and indeed they cited one goal of their study as being for veterinary organizations to recruit and retain staff. Identity was framed as an organizational management tool to “generate competitive advantage through their people by working toward organizational and individual identity congruence” (106).

The studies discussed above were undertaken by researchers who have a close association with the veterinary profession, being either veterinarians or veterinary nurses. By contrast, there are a small number of studies conducted outside the professional practice literature in which veterinarians were selected for research focus by researchers in other scholarly traditions. A sociomaterial ethnographic study conducted with a cultural-studies orientation in the context of a rural veterinary clinic focused on issues of power and cultural capital, finding that material objects were important cultural signifiers, with individuals positioned as veterinarians or as nonveterinary support staff based on their relation to items such as scalpel blades and dirty laboratory equipment (107). A group of organizational studies researchers used a questionnaire with a psychological approach to compare professional identity in veterinarians who were working in clinical and non-clinical settings (108), with findings suggesting that participants who worked in nonclinical settings identified more strongly with their immediate workgroup and profession than with the organization, while employed veterinarians in veterinary medical organizations identified more strongly with their organization and workgroup than with the profession. It is noteworthy that the studies cited here that focus on veterinarians beyond preparation for initial practice are undertaken by social science researchers from outside the veterinary profession.

Professional identity research within the veterinary profession focuses almost exclusively on students and new graduates, with implications being directed to those who educate and prepare undergraduates for practice. While curriculum implications of professional identity development are undoubtedly important, and it seems likely that initial professional education, and the make-or-break period after graduation are critical developmental periods, a position that professional identity development is significant only in the context of students and new graduates, and therefore a matter only for university educators does not seem warranted. Set against a context of rapid social change and multiple professional challenges, veterinary professional identity is salient for all veterinary stakeholders at all stages of the professional trajectory. The elucidation of conceptual frameworks that allow a shifting focus on both individuals and their social, cultural, and material contexts provide the veterinary profession with rich opportunities for rigorous professional identity research. In the following section, we explore some directions and examples in the domain of human medicine.

### 4.2. Professional identity in medicine

Medicine occupies a unique place as the archetype of a profession in the public mind and imagination, and it interfaces with people at some of the most vulnerable and momentous periods of their lives. Their position affords the medical community considerable societal influence and economic reward as well as entailing the obligation on doctors to behave and practice with diligence, expertise, and care. Institutions that support and sustain the profession have long been motivated to understand professional identity and support its development with examples that have explored relations between doctors and the communities they serve, linking professional identity to the notion of a social contract (109) and public trust (110). With clearly defined boundaries of responsibility at all levels from undergraduate student up to specialist doctor, the development of professional identity is presented as a gradual process in which socialization fosters a sense of group membership (111) and the need for supervision and autonomy must be balanced (112).

At an individual level, professional identity research has focused on character in individual doctors, on models of practice and their implications for identity, and on relationships between professionalism and professional identity. In educational settings, the research focus rests on the complex intersections and tensions between learning, development, and professional identity and addresses questions of shared understandings about ethics, responsibility, tradition, behavior, trust, and character (110, 113–118). Furthermore, addressing professional identity can implicitly or openly reinforce or challenge existing assumptions or structures of power, privilege, and vested interests in the medical profession and in the relations between doctors and those with whom they work, whether patients or other health care workers (119, 120). There is much at stake in maintaining a position as a paradigm profession.

Changing times also affect social norms, and demand a radical re-evaluation of tightly held professional traditions and push high status professions like medicine out of taken-for-granted and comfortable positions of privilege. Loss of trust in professionals has been cited as a prompt for professional identity research (121), with some authors strongly arguing for a democratizing agenda in medical education based on transformation of power, identities, and physical locations, with professional identity considered to be an emancipatory tool (122). Power, gender, race, and intersectionality are also themes in the discursive construction of identity (117, 123, 124), with routine clinical activities like clinical rounds presentations representing opportunities to model and learn how talk constructs competence in the development of professional identity in medical students (125). The COVID-19 pandemic has been posited to have impacted on the professional identity formation of medical students as they grapple with a changed learning experience and future (126). The recognition of the powerful influence of context, environment and culture as a shaper of possibilities for professional identity formation is maturing in the medical literature (127) in ways that veterinary researchers have yet to meaningfully engage with.

The intertwinement of the personal and the professional can be viewed uneasily in a profession for which objectivity, certainty, and competence are tightly held as hallmarks of professional position. While outwardly directed benevolence toward their patients and society can be framed in terms of character and stable traits, emotion carries connotations of subjectivity and loss of control. Dealing with patient emotions may be a necessary task for doctors to consider, but the emotions of doctors and medical students have been described as “the ever-present absence” of medical education (128), a phrase that captures a paradoxical sense of discomfort with the centrality of emotion to medicine. Emerging research on emotion and other deeply personal dimensions of medical student learning and professional identity development (114, 116, 129, 130) have advocated “acknowledging the full range of negative to positive emotions and making them an integral and essential part of identity development” (131).

## 5. A conceptual framework for veterinary professional identity

Based on this conceptual analysis, we put forward a narrative conceptual framework that can be articulated as a series of propositions drawing together all the theoretical elements discussed in this paper to characterize professional identity and to contextualize it to the veterinary profession (see Table 2).

**Table 2:** A narrative conceptual framework for professional identity.

• Professional identity development refers to implicit and explicit dialogical and developmental processes of identification that reflect and shape the self and relations with the social world.
• Identity, both individual and collective, is mutually constituted through dialogue with self and others and engagement with the material, economic and political worlds.
• The processes of professional identity development are manifest in the sayings, doings, and relatings of veterinary practice and are formed and enacted over time through the choices veterinarians make in engaging with and coming to an understanding of their professional practice.
• Making meaning of practice through dialogue and narratives is a powerful way in which veterinarian can exercise their agency even, or especially, when they feel most constrained.

The framework highlights the centrality of dialogue and development, the unresolveable intertwinement of the individual with their social, cultural, and material context, and the importance and limitations of individual agency. In social conditions of change, complexity, and uncertainty, engaging in veterinary practice is more than just mobilization of knowledge accompanied by application of technical and professional skills. Through making meaning of their experiences, veterinarians author themselves and their professional practice. Veterinarians are called upon to engage personally and purposefully with meanings that paradoxically, while largely shared, are not given, but must instead be created.

## 6. Concluding discussion

The purpose of this paper has been threefold: firstly, to justify a sociocultural theoretical approach to research in veterinary practice; secondly, to articulate the interdisciplinary theoretical underpinnings of a conceptual framework for veterinary professional identity; and thirdly, to articulate the conceptual framework in narrative terms so that its core concepts and their relations to each other can be understood.

We undertook the first task by highlighting just some of the complex and contested issues and problems facing the contemporary veterinary profession, including challenges of professional status and economics, changing human-animal relations, issues of wellness and feminisation of the profession. We argued that there is a need for research that can bring individual veterinarians and contextual factors into one conceptual frame. The veterinary profession is grounded in a scientific worldview, which has served well for advances in clinical care. However, to understand and explore the veterinary profession and its people, there is a need to extend, enrich, and challenge the individualistic, positivist approach that continues to dominate veterinary professional and scholarly literature. It can be daunting and humbling to step outside one's own professional boundaries to learn from the scholarly traditions of the social sciences.

The body of this paper was dedicated to a detailed articulation of the theoretical underpinnings of a conceptual framework for veterinary professional identity using a socio-cultural lens. The body of the paper concluded with an articulation of the core propositions of the framework. The analysis and framework address a gap in the veterinary scholarly literature that is both practical and theoretical. On the practical level, the framework can be used and drawn upon flexibly for researchers, for practitioners and for the veterinary profession more broadly. The next step of the research is to apply this framework to new veterinary graduates in the exploration of their professional identities. Other researchers could use some or all of the concepts in the framework to guide their own research, broadening their ideas of appropriate research into veterinary practice and professional identity and diversifying notions of research approaches that might be of value. Veterinarians and institutions of the veterinary profession could utilize the concept of veterinary professional identity as a lens on some of the issues and challenges facing the profession, including wellbeing, attrition, economic rewards, and gender. There is insufficient research into how professional identity development is supported and understood in mid- and late career veterinarians, after career breaks and in times of challenge and crisis. On the theoretical level, although the importance of conceptual frameworks has been highlighted in the context of veterinary social science and especially qualitative research (132), there is a paucity of examples grounded in the veterinary context that elaborate and articulate such a rigorous, interdisciplinary conceptual framework. For researchers who are challenged to know what a conceptual framework is and how they might develop and represent their own, this example is likely to be of value–even if their own specific research focus and interests are unrelated to professional identity. The level of detail presented in this paper is certainly not required in every research report exploring veterinary practice. However, those who would conduct rigorous and useful social science research need to use, and to be able to articulate, appropriate guiding conceptual frameworks.

## Author contributions

ES drafted the manuscript and led the development of the conceptual framework. FT supervised the doctoral project, supported the development of the conceptual framework, and reviewed the manuscript. Both authors read and approved the final version.
